# Effectiveness of mHealth Interventions in Medication Adherence among Patients with Cardiovascular Diseases: A Systematic Review

**DOI:** 10.3390/diseases11010041

**Published:** 2023-03-01

**Authors:** Muhammad Arshed, Aidalina Binti Mahmud, Halimatus Sakdiah Minhat, Lim Poh Ying, Muhammad Farooq Umer

**Affiliations:** 1 Department of Community Health, Faculty of Medicine and Health Sciences, Universiti Putra Malaysia (UPM), Serdang 43400, Selangor Darul Ehsan, Malaysia; 2 Department of Dental Public Health, College of Dentistry, King Faisal University, Hofuf 31982, Saudi Arabia

**Keywords:** mobile health, interventions, adherence, cardiovascular diseases medication, patients

## Abstract

mHealth interventions have been reported to improve adherence to long-term therapies in chronic conditions. Therefore, this study aimed at determining the effectiveness of mHealth interventions in medication adherence among patients with cardiovascular diseases (CVDs), a leading cause of mortality globally. Relying on our inclusion criteria and the PRISMA recommendations, a literature search was carried out in the PubMed, Medline, and ProQuest databases for primary studies that investigated the impact of mHealth on medication adherence for cardiovascular disease (CVD) between 2000–2021. A total of 23 randomized controlled trials with 34,915 participants matched the selection criteria. The mHealth interventions used included text messages, mobile phone applications, and voice calls, which were used either as a single intervention or combined. Additionally, studies on enhancing drug adherence had contradictory findings: most of the studies elaborated positive results; however, six studies were unable to reveal any significant effect. Finally, a risk bias analysis revealed varying outcomes across all studies. This review, as a whole, supported the notion that mHealth interventions can be effective in improving adherence to CVD medication even though they could not improve adherence to all CVD medications when compared with controls. Further trials with more refined designs integrated with comprehensive interventions are needed to produce better health outcomes.

## 1. Introduction

Cardiovascular diseases (CVDs) account for 17.9 million deaths annually (31% of all deaths globally) [1]. It is a key contributor to premature deaths and escalating healthcare expenses [2,3]. Cardiovascular diseases are projected by Global Health to remain the leading cause of mortality in 2030 [4]. To reduce the burden of CVDs, adequate control of CVD risk factors is required; such factors include high blood pressure, excess body weight, high blood lipids, cigarette smoking, and diabetes. Elevated blood pressure has been reported to be the most significant contributing factor to CVDs [5]. It was indicated in the Global Burden of Disease study that each of the above-mentioned risk factors was among the top 10 causes of a loss in disability-adjusted life years [6].

Adherence is the degree to which an individual’s behavior in taking medications, maintaining a diet, and implementing other lifestyle changes matches with accepted advice from a healthcare professional. Medication non-adherence is acknowledged as a leading healthcare problem that can be prevented and as a serious obstacle to improving clinical outcomes. Non-adherence to medication represents the leading cause of mortality for more than 60% of cardiovascular patients. Medication use is a complex activity that is influenced by a variety of elements that include experience, beliefs, and culture. Medication-taking habits might vary between different drugs. Faster medical care that boosts medication adherence is currently one of the most serious issues. Despite efforts, randomized controlled trials have only shown a limited impact on medication-taking behavior [7,8,9,10].

Despite substantial resources being allocated to developing new regimens, non-adherence to current medications is persistent and remains an issue of public health importance. This is because non-adherence depletes evidence-based therapy, thereby escalating mortality rates annually and globally and thus contributing to unwarranted healthcare disbursements. The complexity of non-adherence makes it challenging to define and accurately identify its presence in patients, leading to increased risk for worse cardiac events and a higher mortality rate. Therefore, innovative tools for assessing and screening patients for non-adherence will enhance interventions targeted at improving adherence [11]. 

The most widely used interventions to improve cardiovascular medication adherence include unsophisticated pillboxes and calendars, while the Medication Event Monitoring System (MEMS) and blister packs have been used in randomized controlled trials (RCT). Other modern-day interventions commonly used include mobile applications, reminder services, automated dispensers, real-time provider feedback, networkable MEMS, and biomarkers, which can measure adherence objectively [11]. While the availability of these sophisticated tools could be a step in proffering a solution to the issue of non-adherence, their extensive implementation remains restricted. Given that most of the interventions are complicated and not cost-effective, non-adherence behavior necessitates designing and implementing cost-effective interventions.

With the extensive possession of mobile telephones and 5.3 billion mobile broadband global subscribers in 2018 (ICT 2018), the possibility of automation gives rise to the likelihood of designing and implementing cost-effective interventions of behavior changes to a large sample population. In recent times, mobile health is a resource that has gained popularity. The use of mobile phones (mHealth) was reported as a necessity for prompt and enhanced mHealth research and yielded promising results [12]. Targeting behavior modification could be accomplished with the use of mHealth technologies [13].

In addition, mobile phone interventions are associated with only a few adverse events (such as the probability of road traffic accidents) [14]. Even if mHealth is becoming increasingly popular, there is still no proof of its effectiveness [15]. Today, mHealth, electronic health (e-Health), and telehealth can be used as replacements for each other. The Global Observatory for e-Health defined mHealth as the support of medical and public health using mobile devices, which include mobile phones, personal digital assistants (PDAs), patient monitoring devices, and wireless devices.

Even though mHealth interventions to improve medication adherence in chronic disease patients have supposedly been positive, their impact on cardiovascular disease medication adherence is still not clear, so there is an urge to find literature support to establish a causal relationship. Therefore, this systematic review was conducted to determine the effectiveness of mHealth interventions on cardiovascular medication adherence. 

## 2. Materials and Methods

The Preferred Reporting Items for Systematic Reviews and Meta-Analyses (PRISMA) criteria were followed when conducting this systematic review of primary studies on mobile health interventions to increase adherence to cardiovascular medication. By outlining the basic specifications for a protocol, the PRISMA recommendations help authors to improve the reporting of procedures for intended systematic reviews and meta-analyses.

### 2.1. Search Criteria

The target population was patients with the following cardiovascular diseases: hypertension, ischemic heart disease, myocardial infarction, acute coronary syndrome, heart failure, stroke, and peripheral arterial disease. In addition, they had undergone mHealth-led interventions (WHO or ATA definition of mHealth), which were compared with the control or usual care. The outcome of the study was medication adherence as primary or secondary. The search was restricted to a time frame between 2000 (August) and 2021 (July). Only English language studies were reviewed for reporting. The participants’ socio-demographic characteristics (gender, age, location, education level, income, ethnicity, type of client, and years of experience) were not a limitation. 

### 2.2. Inclusion and Exclusion Criteria 

The inclusion criteria were: (1) clinical trials in comparison with the standard of care or control, (2) peer-reviewed journal articles with full text, (3) studies conducted on mHealth interventions to determine the effect on patient adherence to cardiovascular diseases, and (4) only mHealth interventions (WHO or ATA definition of mHealth).

The exclusion criteria were: (1) not original research, (2) not having adherence as the primary or secondary outcome, (3) mHealth interventions not addressing cardiovascular diseases, (4) studies that lacked an appropriate control group or were not randomized, (5) studies that were not in English, and (6) trials that were terminated before their completion.

### 2.3. Search Strategy

This review employed the use of the PubMed, MEDLINE, ProQuest, and Google Scholar databases for an electronic systematic literature search. The standard for Preferred Reporting Items for Systematic Reviews and Meta-Analyses (PRISMA) was applied. The Boolean operators “and” and “or” were used in the search. The grey literature was also researched using a Google Scholar search. The articles were identified by combining Medical Subject Headings (MeSH) with Boolean operators such as: ∗Reminder Systems, ∗Text Messaging; *mobile app; *SMS; *phone call; *digital health [Cardiovascular Diseases [∗prevention & control]; *coronary; *myocard; *cerebrovasc; *stroke; *heart; Cell Phone; and Medication Adherence [∗statistics & numerical data]].

### 2.4. Quality Evaluation

Cochrane’s risk-of-bias assessment tool was used to analyze the studies when evaluating the bias. Moreover, the *Cochrane Handbook for Systematic Reviews of Interventions* was used for the quality assessments of all trials. Allocation concealment, randomization, blinding concealment, attrition rates, sufficient data reporting, and non-selective reporting of outcomes were the markers used for assessing quality. A 2-point Likert scale (Yes (1) and No (0)) was used. Then, studies were categorized into three classes according to their reported score: (1) a score of more than 4, which indicated a good-quality study; (2) a score of 3–4, which indicated a moderate quality; and (3) a score less than 2, which indicated a poor quality.

## 3. Result

**Figure 1:**
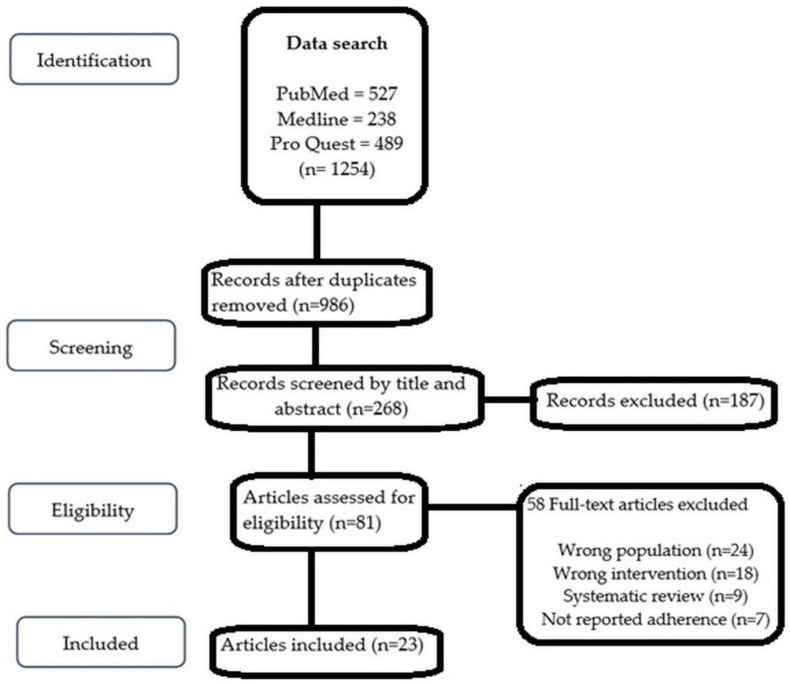
PRISMA (2009) flow diagram showing the flow of search and analysis.

The search of the databases identified a total of 1254 studies that were screened to comply with the eligibility criteria. Studies were eliminated because the content of their titles and abstracts did not meet the inclusion criteria. This systematic review did not include studies that did not use adherence measurement as an endpoint. A good number of studies (58) were excluded due to the wrong populations (participants other than cardiovascular diseases), wrong interventions (interventions other than mHealth intervention), adherence to medication not reported as an endpoint, and for being systematic reviews. Twenty-three peer-reviewed journal articles met the inclusion criteria [16,17,18,19,20,21,22,23,24,25,26,27,28,29,30,31,32,33,34,35,36,37,38] and were taken into account in the analysis. The PRISMA flowchart is depicted in Figure 1.

### 3.1. Characteristics of Trials

This section provides an overview of the authors, the study designs, the intervention subcategories, the types of intervention, the study country, the outcomes, and the adherence measurement tools. Adherence or correlates of adherence were used as the outcome measure. While the study findings provided the final results, the adherence measurement described how adherence was measured in each study. All of these were randomized controlled trials, including seven from the USA with multiple time durations; i.e., one month, 12 months, 28 days, 12 months, 3 months, 6 months, and 3 months, respectively; the sample sizes in these studies were: 90, 21,750, 60, 253, 126, 179, and 413, respectively [17,19,20,21,29,32,33]. Two more studies from Canada had durations of 4 months and 12 months with sample sizes of 90 and 2632, respectively [35,36]. Three studies were conducted in China that were 6 months, 1 month, and 3 months of duration with sample sizes of 280, 50, and 445, respectively [24,31,37]. In addition, two studies were from Pakistan, each with a 3-month duration and with sample sizes of 200 and 201, respectively [23,30]. One more study was conducted in South Africa with a 12-month duration and a sample size of 1372 [25]. Some studies from other parts of world included the following: one from the United Kingdom with a duration of 6 months and a sample size of 301 [18]; one from Australia with a 3-month duration and a sample size of 165 [34]; one from France with a 1-month duration and a sample size of 5546 [16]; one from Malaysia with a 2-month duration and a sample size 62 [22]; one from Iran with a 3-month duration and a sample size of 123 [26]; and one from New Zealand with a duration of 12 months and a sample size of 306 [38] (Table 1).

### 3.2. Types of Intervention

The types of mobile phone-based interventions varied from single SMS interventions [16,17,18,22,23,25,37] to a combination of SMS + Micro Letter (ML) (Micro Letter platforms are open-access Kik Messenger-like programs that provide users in China with access to news and other information) [24]; interactive voice interventions [19]; a multifaceted intervention including medication reconciliation and tailoring; patient education; collaborative care between a pharmacist and a patient’s primary care clinician and voice messaging [21]; interactive text messages [25]; wireless self-monitoring devices [27]; a web-based app using interactive patient assistance tools [28]; individual and peer comparison of reminder alarms [29]; talking treatment intervention that involved SMS and voice calls [30]; WeChat + a BlackBerry reminder app [31]; an alert electronic reminder device (wireless pill bottle) with an automated message sent to the individual via email, text, or automated phone call [32]; and advanced mobile apps [33,34]. One study in Canada used mail-outs and mail-outs plus phone calls [36]; a qualitative study used engagement of intervention and control groups for positive and negative adherence [35]; and Text4HeartII featured educational and motivating materials to promote medication use [38] (as explained in Table 1).

### 3.3. Outcome Measures

Nine trials used the Morisky Medication Adherence Scale-8 (MMAS-8) to measure adherence [17,22,24,27,30,33,34,37,38], one used the Morisky Medication Adherence Scale-4 (MMAS-4) [23], one study used personal inquiry for medication adherence and electronic records [18], one used a modified version of the proportion of days covered [19], one used pillbox bin openings and electronic self-reporting [20], three used the proportion of days covered (PDC) [21,25,36], one used the Hill bone scale [26], one used GlowCap gadgets (electronic pill bottles) [29], one trial used wireless pill bottles [32], one used arachidonic acid-induced platelet aggregation [16], one used three methods (the MMAS-8, Self-efficacy for Appropriate Medication Use (SEAMS), and a Medication Event Monitoring System (MEMS)) [17], one trial utilized missed doses of pills (two) in seven days [28], one trial assessed adherence to medications via a qualitative method that used adherence (positive or negative) [35], and one utilized the MMAS-8 and prescription records to modify behavior [38].

### 3.4. Medication Adherence

The studies found on enhancing drug adherence had contradictory findings: 17 studies (73.9%) reported a significant improvement in medication adherence [16,17,18,19,21,22,23,24,25,26,28,29,32,33,34,35,37], whereas 6 studies (26%) failed to show a substantial impact of the intervention on medication adherence [20,27,30,31,36,38].

### 3.5. Hypertension

In this review, nine studies were found that used blood pressure as an outcome measure along with adherence to medication [18,19,21,23,25,27,31,33,37]. Three trials elaborated a significant decrease in blood pressure and improved medication adherence [25,27,37]. At the same time, six trials could not provide any evidence of a decrease in blood pressure [18,19,21,23,31,33].

### 3.6. Ischemic Heart Disease

Fourteen trials assessed adherence to medication in patients with coronary heart diseases [16,17,19,21,22,24,28,29,30,31,34,35,36,38]. In addition, two trials addressed cardiac rehabilitation after myocardial infarction [35,36]. Ten trials showed a significant improvement in adherence to medication in patients with ischemic heart diseases [16,17,19,21,22,24,28,29,34,35], while four did not reveal any significant improvement.

### 3.7. Heart Failure

In this review, only one study assessed the impact of mHealth on medication adherence in patients with heart failure [20]. This study, which had 60 participants and a 28-day duration, was a four-arm trial with two arms using the ePill box silent and reminder vs. smartphone silent and reminder. No improvement in adherence to medication was documented. 

mHealth has been utilized in patients with peripheral arterial disease. However, in this review, no particular study was found to determine the impact of mHealth on adherence to medication in patients with peripheral arterial disease.

### 3.8. Stroke

Two studies evaluated the impact of mHealth on medication adherence in patients with stroke. They were conducted in Pakistan with sample sizes of 200 and 201 participants and a duration of three months [23,30]. One reported a significant improvement in medication adherence [23], while the other failed to show substantial results [30].

### 3.9. Diabetes Mellitus

One trial evaluated the effect of mHealth intervention on adherence to medication in patients with cardiovascular disease comorbidity of type 2 diabetes mellitus. The trial used three groups: interactive voice recognition phone calls in group 1; interactive voice recognition enhanced phone calls in group 2; and a control group with usual care in group 3. Both intervention groups showed results that were 2.2 percentage points higher than those of the control group (95% CI, 1.1–3.4) with a difference of 3.0 (95% CI, 1.9–4.2) [19].

### 3.10. Risk of Bias and Methodological Quality Assessment

**Figure 2:**
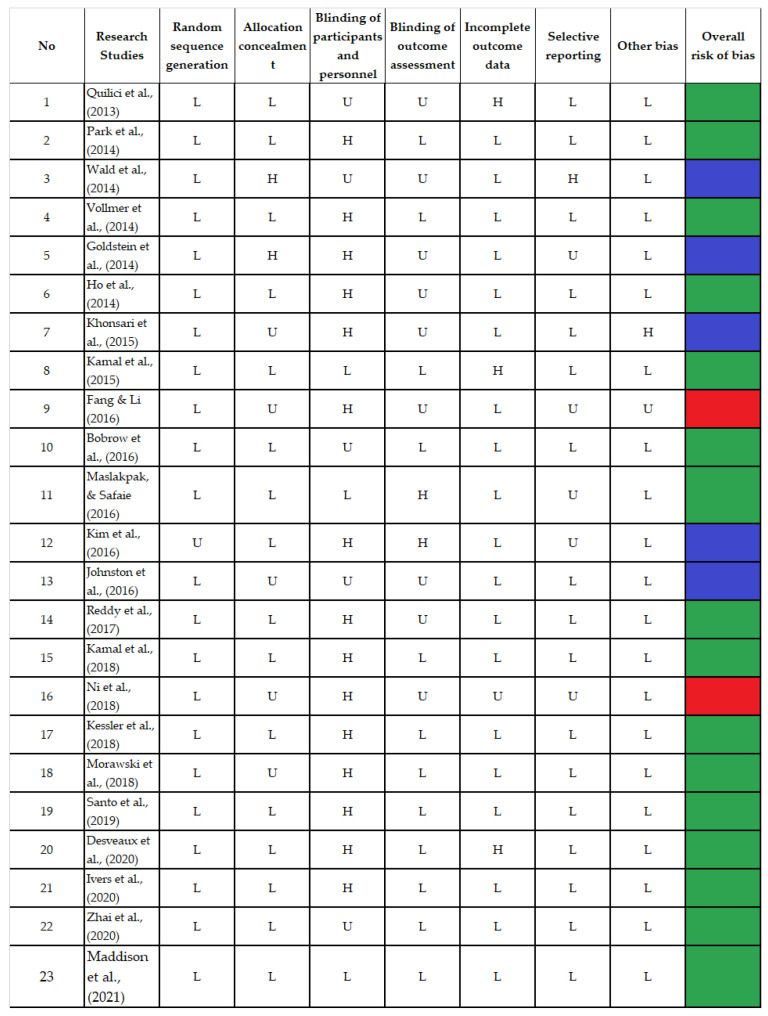
Bias Risk Assessment [16,17,18,19,20,21,22,23,24,25,26,27,28,29,30,31,32,33,34,35,36,37,38].

Seven domains were scored as low, high, and unclear risk of bias. Fifteen trials were considered high-quality studies because they received good scores across at least five domains [17,19,21,23,25,26,29,30,32,33,34,35,36,37,38], while six trials achieved good scores in less than five with a moderate quality [16,18,20,22,27,28], and two received good scores in only two of the seven domains and had a poor quality [24,31]. The bias in the included studies was systematically assessed and is presented in Figure 2.

## 4. Discussion

In recent times, technology-based interventional approaches such as mHealth, eHealth, and telehealth have become essential tools. mHealth technology is at the same time assessable, faster, and acceptable to the community.

Findings from previous studies showed improvement in medication adherence in patients after mobile health interventions. mHealth, which has gained popularity in recent times, has attained amassed interest as a tool for enhancing health promotion interventions as well as the provision of CVD prevention in an accessible manner with a relatively lower cost. Moreover, mHealth has a specific potential to promote lifestyle modification and is an effective tool for improving adherence [39].

In the present review, studies on enhancing drug adherence had contradictory findings: 17 trials (73.9%) reported a significant improvement in medication adherence, whereas 6 studies (26%) failed to show a substantial impact of the intervention on medication adherence. The effectiveness of mHealth interventions on cardiovascular medication adherence has also been reported in other systematic reviews, which showed that the majority of studies reported an improvement in medication adherence and other health outcomes [39,40]. Similar findings from an existing body of literature strengthen the current review’s evidence [41,42,43].

In the current review, most of the studies used SMS only as an intervention, while some trials used SMS in combination with Micro Letters or voice calls. In a meta-analysis review, patients who received SMS-based interventions were found more likely to be adequately adherent than the control group [44].

mHealth apps (mobile and web-based) were also another popular mHealth intervention identified in the current review. A review was conducted to assess the impact of mHealth apps on adherence to CVD that revealed mixed results with reasonable acceptability [45]. As such, the two commonly used modes of mHealth delivery are text messaging (short messaging service or SMS) and smartphone applications [39]. In the present review, seven studies employed the use of SMS to improve medication adherence, while five studies employed mHealth-based apps. Both SMS and phone applications have the ability to serve the role of a drug intake reminder, blood pressure monitor, or routine clinic appointment reminder [40]. 

The current review identified other mHealth technologies used that included phone calls, interactive patient support apps, interactive text messages, interactive voice calls, wireless self-monitoring gadgets, electronic pill bottle reminder devices, emails, automated messages, and phone calls. Similarly, numerous effective delivery strategies were identified in other systematic reviews; these included SMS [46,47], automated phone messages, and reminders [48].

However, in certain cases, the reported beneficial outcome was not always a result of the patient’s choice of a particular tool. A study compared different interventions to determine their effects on adherence to medication. The study used various tools that included pillboxes and smartphones. Smartphones reported a higher adherence score than pill counts [20]. Moreover, a multifaceted intervention medication reconciliation and tailoring, patient education, collaborative care between a pharmacist and a patient’s primary care clinician, and voice messaging were used [21].

A meta-analysis of mHealth intervention to improve adherence in chronic diseases such as diabetes mellitus, asthma, cardiovascular diseases, epilepsy, and HIV infection showed improvement in medication adherence; this included 16 randomized controlled trials [49]. mHealth intervention had a positive impact on improving antiretroviral therapies in HIV patients and in antituberculosis treatment [49]; however, cardiovascular diseases require further refined designs.

mHealth is a more personalized method that helps individuals in managing their condition using an apt and standard design form. It has also been found to be an efficient tool in various studies of real-time data analysis. A study showed interest in smartwatches because they are a potential tool and have multifunctional dimensions like a smartphone. The study presented a smartwatch-based medication reminder system and also introduced an early prototype to improve medication adherence [50]. Furthermore, it could help the clinicians to improve adherence to treatment, which could be beneficial to the the patient [51]. This has been found in different studies in which medication adherence was measured using different designs, sample sizes, intervention approaches of mHealth, adherence measuring tools, outcome measures, and the multitude of methods.

**Figure 3:**
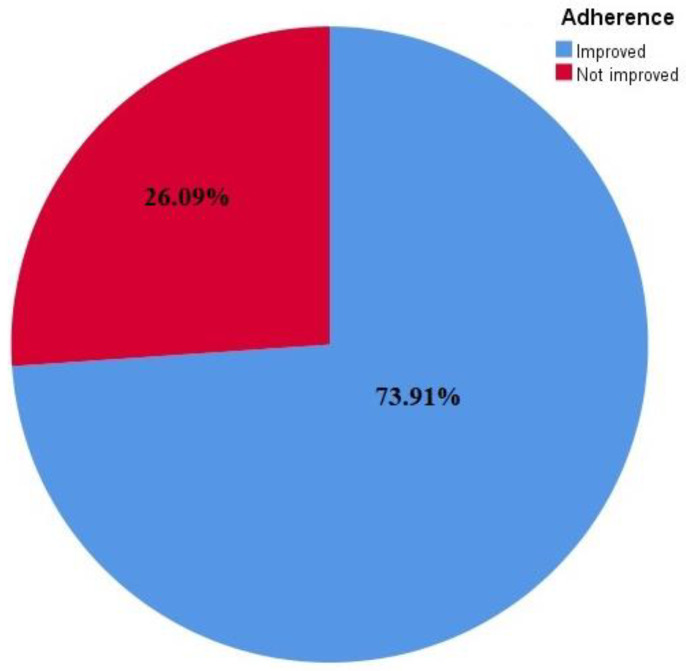
Adherence improvement in response to interventions reflected in the studies included.

Mobile health strategies are instrumental in addressing the issue of medication non-adherence, which has been a challenge in the management of chronic diseases. Despite the positive feedback reported regarding the effectiveness of mHealth interventions in adherence to cardiovascular medications, more studies on the cost-effectiveness of this intervention type are required, especially in resource-limited settings. Some studies have stated that mHealth interventions are cost-effective; for instance, an mHealth intervention study in Kenya reported a marginal cost of USD 0.02 per SMS and a weekly SMS, which represented a cost of almost USD 1 per patient annually. This suggested that mHealth intervention requires a minimum cost to implement but has the potential to yield effective results. However, it was suggested that the cost of other equipment used (as well as the training of research assistants) be included in the total cost. Apart from being less capital intensive, implementing this type of intervention is also convenient for both the health provider and the patient who is the recipient because messages reach the patient at any time and place. This intervention is also less time-consuming and curbs the problem of accessibility to the health facility because patients can communicate directly with their health providers through voice calls and phone apps. It is convenient in resource-limited facilities where venues for routine health education sessions are limited or unavailable, but health information can be delivered through this medium. The participants in all of the chosen trials were diverse. The fact that they varied from one another in terms of their medical and social profiles may have influenced the results, but the authors used the data from the studies to assess how well the interventions worked. Additionally, there was no established time frame for assessing therapies because adherence is a behavior that lasts a lifetime. Studies on enhancing drug adherence have contradictory findings, where seventeen studies (73.91%) reported significant improvement in medication adherence whereas, six studies (26.09%) failed to show a substantial impact of the intervention on medication adherence. Overall, RCTs using various interventions in the current review showed improvement in adherence to CVDs (Figure 3).

However, in planning a mHealth intervention, it is recommended that studies of this nature design an acceptable, standardized, systematic, and validated mobile phone application to facilitate the implementation. In the present review, only the studies in [34,52] had customized applications designed for their interventions. Furthermore, the timing of sending messages and making calls as well as the language used must be acceptable to and understandable by the patients. Close attention should be paid to the content and frequency of the messages and calls to fully achieve the goal of the intervention and gain the benefits thereof. An alternative method should be considered for patients who have problems with their sight, speech, or hearing, as well as for patients who cannot read. All these factors should be considered in designing mHealth interventions. Lastly, one of the goals of behavioral interventions is to produce lasting or durable behavioral changes that will yield positive health outcomes. On this note, it is suggested that future research on mHealth focus on the long-term impact of this intervention on adherence behavior among cardiovascular disease patients because this will provide more authentic findings and justifications.

## 5. Conclusions

This review, as a whole, supported the notion that mHealth interventions can be effective in improving adherence to CVD medication even though the use of various mHealth interventions could not improve adherence to all CVD medications when compared with controls. Further trials with more refined designs integrated with comprehensive and effective interventions are needed. Furthermore, cost-effectiveness studies of such interventions should be conducted to further derive the benefits of mHealth interventions. This will enable policymakers to reserve capital-intensive interventions for patients who are most in need of such interventions; for instance, patients dealing with factors that mHealth cannot overcome (such as adverse drug reactions or a high pill burden).

## Figures and Tables

**Table 1:** Summary of impacts of a mobile health intervention on medication adherence among patients with cardiovascular diseases.

No.	Authors	Study Design	Country	Population	Adherence Measurement	Study Intervention	Outcomes		Findings
							Primary	Secondary	
1	Quilici et al. (2013) [16]	RCT (1 month)	France	*n* = 5546 ACS patients after PCI on aspirin medication	AA-Ag	Two-arm trial: Arm-1: daily SMS reminders for aspirin-adherence in intervention arm; Arm-2: control (no intervention)	Adherence	Not mentioned	Adherence to aspirin improved in the intervention arm with SMS reminders with an odds ratio of 0.37 to 0.02.
2	Park et al. (2014) [17]	RCT (1 month)	USA (Northern California)	*n* = 90 ACS patients	1—MMAS-8 2—SEAMS 3—MEMS	Three-arm trial: Arm-1: reminder text messages for medication; Arm-2: rext messages for education; Arm-3: control (no intervention)	Adherence	Patient feasibility and satisfaction with the text messages	Text messaging intervention improved adherence. Patients with text message interventions showed a higher percentage of accurate doses (*p* = 0.02), percentage of doses taken (*p* = 0.01), and percentage of prescribed doses taken on time (*p* = 0.01). Antiplatelet response rates were higher than statin response rates (*p* = 0.005) as per the schedule.
3	Wald et al. (2014) [18]	RCT (6 months)	UK (London)	*n* = 301 Patients on AHT + lipid-lowering medication	1—Personal inquiry for medication adherence 2—Electronic records for drug prescription	Two arm trial: Arm-1: text messaging intervention for reminders to take medication; Arm-2: control (no intervention)	Adherence	Blood pressure and serum cholesterol	Adherence significantly improved compared to the control group. Non-adherence to medication was lower among the text messaging group (14/150 (9%)) than in the control group (38/151 (25%)). No statistically significant differences in blood pressure were found.
4	Vollmer et al. (2014) [19]	RCT (12 months)	USA (Northwest, Hawaii, and Georgia)	*n* = 21,752 CAD patients with type 2 diabetes (suboptimal adherence to medication)	Modified version of the PDC	Three-arm trial: Arm-1: phone calls (interactive voice); Arm-2: interactive voice-recognition-enhanced phone calls; Arm-3: control (no intervention)	Adherence	Blood pressure and lipid levels	Adherence significantly improved in both intervention groups. Both intervention groups showed 2.2 percentage points higher than the control group with odds ratios of 1.16 and 1.14 higher in the first and second intervention groups than the control.
5	Goldstein et al. (2014) [20]	RCT (28 days)	USA (Ohio)	*n* = 60 Patients with CF	Pillbox bin openings were used for the telehealth intervention group and electronic self-reporting for people with them—health intervention	Four-arm factorial feasibility trial: two arms with pillbox silent, pillbox reminding; and two arms with smartphone silent, smartphone reminding	Adherence	Acceptance of model	No improvement in adherence. The total adherence rate was 78% (SD 35), with the telehealth device adhering 80% of the time and people with a smartphone adhering 76% of the time; reminders adhered 79% of the time while reminding did not improve adherence.
6	Ho et al. (2014) [21]	RCT (12 months)	USA (Colorado, Washington, North Carolina, and Arkansas	*n* = 253 ACS/MI patients	PDC > 0.8	Two-arm trial: Arm-1: multifaceted intervention, medication reconciliation and tailoring, patient education, collaborative care between a pharmacist and a patient’s primary care clinician, and voice messaging; Arm-2: control	Adherence	Blood pressure (BP) and low-density lipoprotein cholesterol (LDL-C)	Adherence improved. The intervention group showed 89.3% adherent patients vs. 73.9% in the usual care group (*p* = 0.003). In addition, the intervention arm had a greater mean proportion of days covered, with 0.94 vs. 0.87 (*p* = 0.001). No significant decrease in systolic blood pressure was found.
7	Khonsari et al. (2015) [22]	RCT (2 months)	Malaysia	*n* = 62 ACS patients	MMAS-8	Two-arm trial: Arm-1: automated SMS reminders; Arm-2: control (no intervention)	Adherence	Heart functional status	Adherence was found to improve by (χ(2) (2) = 18.614, and heart functional status was also improved.
8	Kamal et al. (2015) [23]	RCT (3 months)	Pakistan	*n* = 200 Post-stroke patients treated for high blood pressure	MMAS-4	Two-arm trial: Arm-I: SMS for reminders to take medication; Arm-2: control (no intervention)	Adherence	DBP	Adherence improved by 7.4 in the intervention group and 6.7 in the control group, while the mean diastolic blood pressure in the intervention group was 2.6 mmHg lower vs. the control group.
9	Fang and Li (2016) [24]	RCT (6 months)	China	*n* = 280 CAD patients post-angiography /CT	MMAS-8	Three-arm trial: Arm-1: utilized SMS; Arm-2: utilized SMS + Micro Letter, Arm-3: control with the phone only	Adherence	Not mentioned	Improved adherence (improved score not mentioned).
10	Bobrow et al. (2016) [25]	RCT (12 months)	South Africa	*n* = 1372 Hypertensive patients	Proportion of days calculated + EuroQol Group 5-Dimension Self-Report Questionnaire	Three-arm trial: Arm-1: text message reminders for medication adherence; Arm-2: interactive text messages; Arm-3: control (usual care with no texts)	SBP Control	Adherence and quality of life	Improved adherence; odds ratio for PDC was 1.86 (*p* < 0.001) for information-only messaging vs. usual care and 1.60 (*p* = 0.002) for interactive messaging vs. usual care. SBP was significantly reduced in both experimental groups; the mean change for the information-only message group vs. the usual care group was −2.2 mm Hg (*p* = 0.046) and for the interactive message group vs. usual care group was −1.6 mm Hg (*p* = 0.16).
11	Maslakpak and Safaie (2016) [26]	RCT (3 months)	Iran	*n* = 123 Hypertensive patients	Hill-Bone Medication Adherence Scale	Three-arm trial: Arm 1: SMS; Arm 2: reminder card; Arm 3: control (no intervention)	Adherence	Not mentioned	Improved adherence; SMS (57.70 ± 2.75), reminder cards (57.51 ± 2.69), vs. control (46.63 ± 2.99) (*p* < 0.001).
12	Kim et al. (2016) [27]	RCT (6 months)	Republic of Korea	*n* = 95 Hypertensive patients	MMAS-8	Two-arm trial: Arm 1: wireless self-monitoring app; Arm-2: control (no intervention)	Adherence, PAM, SBP, and DBP	Not mentioned	No improvement in adherence. Improved SBP (beta = −0.27, *p* = 0.02) and DBP (beta = −0.34, *p* = 0.007) with patient activation.
13	Johnston et al. (2016) [28]	RCT (6 months)	Sweden	*n* = 174 Post-MI patients	2 missed doses throughout a maximum 7-day observation cycle	Two-arm trial: Arm-1: interactive patient assistance tool (web-based software); Arm-2: control	Non-adherence score	Change in cardiovascular risk factors and quality of life	Adherence improved. Score for non-adherence: intervention vs. control (16.6 vs. 22.8) (*p* = 0.025). In the intervention group, patient satisfaction was also higher.
14	Reddy et al. (2017) [29]	RCT (3 months)	USA (Philadelphia)	*n* = 126 30–75 years old, 65 years old, 96% male patients with CAD with poor adherence	GlowCap (electronic pill bottle)	Three-arm trial: Arm-1: feedback from a participant who received a daily alarm and a weekly report on their medication adherence; Arm-2: feedback from a partner who also received an alarm and a weekly report that was shared with a friend, family member, or peer; Arm-3: control	Adherence	Adherence (post-intervention) change in LDL and patient activation	Adherence improved in both intervention arms compared to control. Individual feedback arm: 89%; partner feedback arm: 86%; control arm: 67%; (*p* < 0.001).
15	Kamal et al. (2018) [30]	RCT (3 months)	Pakistan	*n* = 201 CAD + CVA patients	MMAS-8	Two-arm trial: Arm-1: daily interactive voice call, daily medication reminder, and weekly lifestyle modification messages; Arm-2: control (no intervention)	Adherence	Not mentioned	Adherence improved insignificantly: 7.41 in the intervention group vs. 7.38 in the control group. However, it was not statistically significant (*p* = 0.40).
16	Ni et al. (2018) [31]	RCT (1 month)	China	*n* = 50 CHD patients	1—Number of dosages taken by a patient 2—Voils Medication Non-Adherence Extent Scale 3—Likert scale of 5 points	Two-phase, two-arm trial: We Chat, BB reminder app vs. controls (no intervention)	Adherence	SBP and DBP	Adherence improved insignificantly; non-adherence reduced to −1.35 in the experimental group vs. −0.69 in the control group. However, it was not statistically significant (*p* = 0.33). The SBP was reduced by 3.76 in controls vs. an increase of 0.93 in the experimental arm but this was statistically insignificant (*p* = 0.51). DBP was decreased significantly.
17	Kessler et al. (2018) [32]	RCT (6 months)	USA (Philadelphia)	*n* = 179 Age: 18, 52 y; 65% male CVS Health employees or their dependents with active CVS Caremark prescription coverage	Wireless pill bottle opening	Four-arm trial: Arm-1: medication adherence partner (friend/family); Arm-2: alert reminder (wireless pill bottle) + automated message (email, text, or automated phone calls); Arm-3: alert and partner; Arm-4: control	Adherence	Not mentioned	Adherence improved. Alert arm: 52.9% vs. controls: 17.0% (*p* = 0.002); partner + alert arm: 54.5% vs. control: 18.6%, 95% (*p* = 0.003).
18	Morawski et al. (2018) [33]	RCT (3 months)	USA (California)	*n* = 413 Self-reported hypertension	MMAS-8	Two-arm trial: Arm-1: given a mobile app intervention; Arm-2: control (no intervention)	Adherence	SBP	Improved adherence; statistically significant change in mean medication adherence between the smartphone app and control group (difference: 0.4; *p* = 0.01); while no significant change in blood pressure was noted (difference: −0.5; *p* = 0.78).
19	Santo et al. (2019) [34]	RCT (3 months)	Australia	*n* = 165 CHD patients	MMAS-8 and PDC	Three-arm trial with the usual care arm, basic medication reminder app, and advanced medication app	Adherence	Adherence according to PDC, blood pressure, and cholesterol	Adherence improved concerning each medicine. The app received a response from 95% of the patients.
20	Desveaux et al. (2020) [35]	RCT (4 months)	Canada	*n* = 90 Post-MI patients	Adherence (positive and negative outcomes)	Three-arm trial: Arm-1: positive adherence as an endpoint; Arm-2: negative adherence as an outcome; Arm-3: did not involve, with negative adherence as an outcome.	Adherence	Not mentioned	The intervention facilitated adherence.
21	Ivers et al. (2020) [36]	RCT (12 months)	Ontario, Canada	*n* = 2632 Adults; 67 y; 70% male with CA after MI discharged from CRC	PDC	Three-arm trial: Arm-1: mail-outs; Arm-2: mail-outs plus automated phone calls; Arm-3: control (usual care)	Adherence + completion of cardiac rehabilitation	Not mentioned	No improvement in adherence. Medication adherence odds ratio: 1.02 (0.78–1.32) (*p* = 0.91); mail-outs: 0.95 (0.68–1.10) (*p* = 0.73); mail-outs/calls Statin adherence (PDC = 0.8): 0.89 (0.69–1.16) (*p* = 0.39); mail-outs: 1.04 (0.75–1.30). However, mail-and-phone interventions could boost cardiac rehabilitation completion after myocardial infarction.
22	Zhai et al. (2020) [37]	RCT (3 months)	China	*n* = 445 Hypertensive patients	MMAS-8	Two-arm cluster RCT: Arm-1: SMS-based reminders on adherence to medication vs. control (no SMS)	SBP DBP	Adherence	Improved adherence. Mean medication adherence: 7.4 in the intervention group vs. 7.0 in the control group (*p* = 0.04); while SBP was reduced and showed a mean SBP of 134.5 mm Hg in the intervention group vs. 140.7 mm Hg in the control group (*p* = 0.001).
23	Maddison et al. (2021) [38]	RCT (12 months)	New Zealand	*n* = 306 Participants with ACS/MI/percutaneous coronary revascularization	Prescription records, MMAS-8, A modified behaviour score (European Prospective into Cancer–Norfolk prospective population research)	Two-arm trial: Arm-1: Text4HeartII intervention; Arm-2: control (no intervention)	Adherence to medication at 24 weeks	Adherence to medication ratio at 52 weeks	No improvement in adherence. Medication adherence in intervention group vs. usual care (87/153 (56.8%) vs. 105/153).

RCT: randomized control trial; SBP: systolic blood pressure; DBP: diastolic blood pressure; SMS: short message service; PDC: proportion of days covered; MMAS-8: Morisky Medication Adherence Scale-8; MMAS-4: Morisky Medication Adherence Scale-4; ACS: acute coronary syndrome; MI: myocardial infarction; AA-Ag: arachidonic acid-induced platelet aggregation; CAD: coronary artery disease; CHD: coronary heart disease; CR: cardiac rehabilitation; CRC: cardiac rehabilitation center; CF: cardiac failure; MEMS: Medication Event Monitoring System; PCI: percutaneous coronary intervention; SEAMS: Self-efficacy for Appropriate Medication Use.

## Data Availability

All accessed data for this study is shared in the manuscript. In case of any further requirement, 1st author can be accessed.
